# A question prompt sheet for adult patients with chronic kidney disease

**DOI:** 10.1186/s12882-016-0362-z

**Published:** 2016-10-19

**Authors:** Swati Lederer, Michael J. Fischer, Howard S. Gordon, Anuradha Wadhwa, Subhash Popli, Elisa J. Gordon

**Affiliations:** 1 Center of Innovation for Complex Chronic Healthcare, Edward Hines Jr. VA Hospital, Hines, IL USA; 2 Jesse Brown VA Medical Center, Chicago, IL USA; 3 Department of Medicine, University of Illinois at Chicago College of Medicine, Chicago, IL USA; 4 Department of Medicine, Edward Hines Jr. VA Hospital, Hines, IL USA; 5 Loyola University Medical Center, Maywood, IL USA; 6 Center for Healthcare Studies, and Department of Surgery, Division of Transplantation, Northwestern University Feinberg School of Medicine, 633 N. St. Clair, 20th Floor, Chicago, IL 60611 USA

**Keywords:** Patient-centered care, Kidney disease, Shared-decision making

## Abstract

**Background:**

Patients with chronic kidney disease (CKD) commonly have unmet information needs. Greater patient participation in healthcare discussions can address these needs and improve health outcomes. We developed a patient-centered question prompt sheet (QPS) to engage CKD patients in healthcare conversations.

**Methods:**

We conducted a two phase, mixed-methods, cross-sectional study involving semi-structured telephone interviews. Patients with an estimated glomerular filtration rate (eGFR) <60 ml/min/1.73 m^2^, on dialysis, or with a kidney transplant were recruited from one Veterans Affairs (VA) nephrology clinic. Phase 1 interviews included open-ended questions assessing patients’ CKD-related information needs and generated a preliminary 67-item QPS. Phase 2 interview participants rated the importance of asking each question on a 5-point Likert scale and provided open-ended feedback. All participants rated their willingness to use a CKD-QPS. Input from patient ratings, a multidisciplinary team, and from members of the National Kidney Disease Education Program (NKDEP) Coordinating Panel helped to shorten and refine the QPS. A qualitative thematic approach was used to analyze open-ended responses. Quantitative data were analyzed for means and proportions.

**Results:**

Eighty-five patients participated. Most were male (97 %), non-Hispanic white (71 %), and mean age was 67 years. Patients desired more information about CKD, particularly dialysis/transplant, and the relationship between CKD and comorbid medical conditions. The final QPS included 31-questions divided into 7 CKD subtopics. Most patients (88 %) reported being ‘completely’ or ‘very’ willing to use a CKD-QPS in future doctor visits.

**Conclusions:**

CKD patients have unmet information needs. We developed a QPS to engage CKD patients in healthcare discussions and to facilitate patient-centered care. Future research should assess whether the CKD-QPS addresses patients’ information needs, enhances doctor-patient communication, and improves health outcomes.

**Electronic supplementary material:**

The online version of this article (doi:10.1186/s12882-016-0362-z) contains supplementary material, which is available to authorized users.

## Background

Chronic kidney disease (CKD) is associated with poor health outcomes including risk of end-stage kidney disease (ESKD), cardiovascular disease, and death, and affects approximately 14 % of adults in the United States [1]. Interventions that engage patients in their CKD care can improve disease-related outcomes and are critically needed. Encouraging patients to take an active role in healthcare conversations and disease management is an essential component of patient-centered care [2]. Patients’ involvement in healthcare conversations can increase their knowledge, improve their engagement in self-care practices, motivate adherence to recommended CKD treatment, and attenuate CKD progression [3–5].

Because inadequate communication between CKD patients and their providers persists [6–8], many patients have difficulty comprehending the impact of CKD on their life [7–9]. Accordingly, patients’ unmet information needs may limit their ability to manage their disease and participate in shared decision-making [7–9]. Education about managing CKD is an important component of CKD patient-provider communication [10]. CKD patients commonly exhibit passive communication behaviors (e.g., do not ask questions) [6, 11]. Studies have attributed chronically ill patients’ passivity during healthcare encounters to various factors including: disease-related anxiety, inadequate disease knowledge, not knowing what questions to ask, and trust in doctors to provide necessary information [6, 12].

An effective strategy to foster active patient communication entails use of a question prompt sheet (QPS) [13–15]. A QPS is a list of prepared questions that patients can review prior to their healthcare visit to select the questions that address their specific information needs. QPSs have been developed in other chronically ill populations to stimulate meaningful patient-provider dialogue and are well-received by patients [15–17]. Most QPS research has focused on the cancer patient population; however, QPSs have been developed for primary care patients, parents of children with attention deficit-hyperactivity disorder, and for surgical consultations [18–20]. No QPSs have been developed for CKD patients. Question-asking may be particularly effective for improving CKD outcomes because the disease is typically asymptomatic until renal replacement therapy is indicated. Thus, patients may not be prepared when providers initiate conversations pertaining to CKD treatment options. Most interventions to facilitate CKD patient-provider communication focus on improving providers’ delivery of information rather than on directly activating patients [11, 21]. The objective of this study was to create a QPS for patients with moderate CKD based on their reported information needs.

## Methods

We used a 2-phase, mixed-methods, cross-sectional approach with separate patient cohorts to create a CKD-QPS. We modeled our QPS development on QPS interventions in other chronically ill patient populations [19, 22, 23], by obtaining both patient and expert opinion. Our intention was to create a 30–35 item QPS, comparable to the mean number of questions in other studies [13].

### Participants and settings

Eligible participants were at least 18 years of age, English-speaking, with moderate to advanced CKD (eGFR <60 ml/min/1.73 m^2^), receiving chronic dialysis, or with a kidney transplant and had visited the outpatient nephrology clinic at the Edward Hines, Jr. United States Veterans Affairs (VA) Hospital between April 1^st^ and October 31^st^, 2014. Though the target population for the QPS was patients with moderate disease, those with more severe CKD were included to obtain valuable insight about information patients wished they had known earlier and because a significant portion of CKD patients are referred to nephrologists with advanced disease [24]. During phase 1, the eligible patient pool was stratified by race, ethnicity and gender to oversample for women, African Americans, and Hispanics, allowing for representation of their CKD information needs. The pool of women and minority patients was exhausted early on in phase 2, limiting further stratification.

The Modification of Diet in Renal Disease equation estimating glomerular filtration rate (eGFR) was used to classify CKD according to conventional CKD stages [25]. Patients who were cognitively impaired (Six Item Screener) [26], unaware of their CKD diagnosis, or participating in another VA study were excluded. Eligible participants were mailed an information sheet describing the study and then received a telephone call one week later to screen for eligibility. The Hines VA Institutional Review Board (IRB) approved the study. All participants provided verbal informed consent.

### Phase 1 data collection

In Phase 1, semi-structured telephone interviews were conducted to identify patients’ CKD information needs, and to begin formulating questions that patients believed were important to ask providers. Interviews included 17 open- and 15 closed-ended questions, as previously described (Additional file 1) [6]. Open-ended questions assessed overall CKD information needs, as well as probed for 9 specific domains of CKD care (e.g., diagnosis, cause, disease progression, management, prevention, self-care practices, relationship between CKD and comorbid conditions, complications, and treatment options). Another open-ended question regarding the optimal time for dialysis and transplant education was asked to assess patients’ preference for timing of this important discussion. One female nephrologist trained in qualitative research (S.L.) conducted all interviews. Given that patients provided verbal consent via telephone instead of written consent, the IRB did not permit audio-recording of the telephone interviews. Thus, hand-written notes were taken that represented patients’ responses verbatim and/or through close paraphrase and then converted into transcriptions of the interview dialogue, as is standard practice in qualitative research methods [27]. Mean interview time was 39 min (range: 19–74 min).

### Phase 1 data analysis

**Fig. 1:**
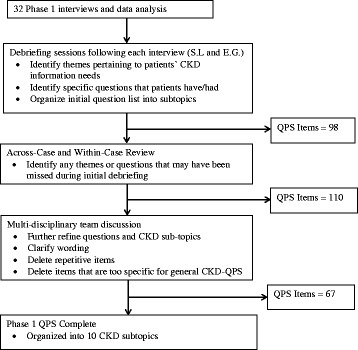
Phase 1 Data Analysis Process

Qualitative data (e.g., transcriptions of interviewee responses to open-ended questions) were analyzed using a thematic approach. After completing each interview, two investigators (S.L. and E.G.) routinely debriefed to identify emerging themes pertaining to patients’ CKD information needs and to register patients’ specific CKD questions into a temporary item bank. The process of developing the 67-item QPS is depicted in Fig. 1.

The debriefing process led to identification of 98 patient questions that were organized into CKD domains of care. Domains and questions were refined using an inductive, thematic approach to analyze participant responses. Thematic analysis entailed searching interview transcripts for repetition and patterns of key concepts and terms [28]. Next, all interview transcripts were re-examined as individual files (within-case) and as a list of all participant responses to each open-ended question (across-cases) to ascertain any questions or themes that might have previously been missed [29]. An additional 12 questions were identified during this process. We adapted language from published QPSs to prepare two different introductory paragraphs, explaining the QPS purpose and instructions on its use [22, 30].

The 6-person multidisciplinary research team comprised of four nephrologists, one internist and health communication expert, and one social scientist reviewed the QPS draft to improve organization, clarify question wording, and remove items that were repetitive or too-narrowly focused for the general CKD population. After refinement, the QPS included 67 questions. Several repetitive items were deliberately retained to assess patient preference in wording in Phase 2. Means and proportions for closed-ended questions were calculated with SPSS version 22 (Chicago, IL USA) in both phases.

### Phase 2 data collection

In Phase 2, semi-structured telephone interviews were conducted to refine and reduce items from the phase 1 QPS. The QPS was mailed to a new pool of eligible patients. During the interview, participants were asked to rate the importance of asking each QPS question on a 5-point Likert Scale, anchored by ‘not at all’ and ‘entirely’, and to obtain open-ended feedback on all items. Open-ended feedback was obtained to assess the clarity of question wording, suggestions for improving wording, preference between repetitive questions, input on the overall list organization, and suggestions for additional questions to add or questions to cut. Participants also provided their preference between the two introductory statements and for one comprehensive QPS or different, albeit overlapping QPSs tailored to CKD severity (e.g., general CKD, dialysis and kidney transplant). Fifteen closed-ended questions assessed patients’ willingness to use a CKD-QPS and self-reported demographic characteristics. Two female interviewers (S.L., H.K.) trained in qualitative data collection and without previous encounters with participants conducted the interviews. Patients’ question ratings and open-ended responses were documented with verbatim handwritten notes. Mean interview time was 52 min (range: 19–122 min).

### Phase 2 data analysis

**Fig. 2:**
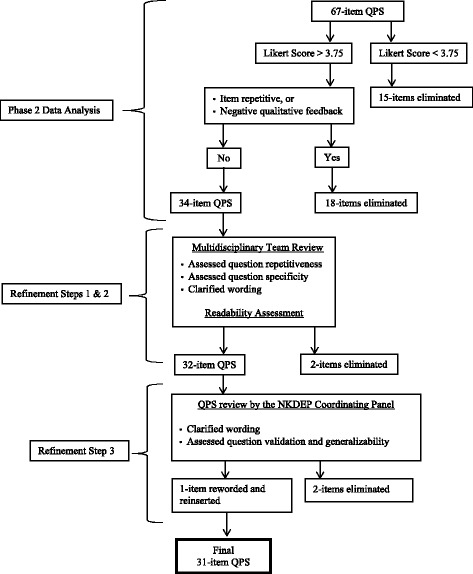
Phase 2 QPS Data Analysis and Refinement Process

Phase 2 QPS development is depicted in Fig. 2. Qualitative and quantitative data were analyzed together. Means for each item’s Likert score were generated. Open-ended responses were analyzed in an iterative manner using the same thematic approach as described above, until reaching saturation. Debriefing sessions and data collection occurred concomitantly, and newly generated questions were asked in subsequent interviews. For example, many patients reported finding several preliminary items repetitive; therefore, subsequent participants were asked which of those questions they most preferred.

Three key members of the multidisciplinary team, 2 nephrologists and 1 social scientist, met once to perform interim analyses for quality assurance, and assess for adequate participant representation and data saturation [31]. No questions were eliminated at that time. The three investigators agreed that data saturation occurred after approximately 40–45 interviews, but additional interviews were conducted to confirm patients’ preferences.

Regardless of CKD severity, participants generally rated all questions highly (e.g., ‘very’ or ‘entirely’ important), which limits the usefulness of mean ratings, underscores the importance of qualitative feedback, and limits the ability to perform meaningful CKD severity subgroup analyses. Generally, items were cut when they scored less than a mean of 3.75 because those items all generated primarily negative patient responses. This resulted in the elimination of fifteen items. Among items scoring above 3.75, eighteen items were removed from the list because the item received negative patient responses or was repetitive with a more preferred question.

Examples of repetitive questions included: ‘What food should I eat?” versus “What food should I avoid?” Patients preferred items on what to avoid rather than the alternate. Examples of negative comments resulting in item elimination included: the answer to the question was obvious (e.g., “Why do you take blood and urine tests so often?”), the item was too specific (e.g., “What is the difference between an AVF, an AVG, and catheter?”), the question undermined providers’ management (e.g., “Do any of my medications or doses need to be changed?”), and that providers cannot offer a meaningful response (e.g., “Did I do something to cause kidney disease?”). If two repetitive questions were equally liked by patients (e.g., “What happens if I do not do dialysis or transplant” versus “Will I die if I do not do dialysis or kidney transplant?”), we retained the question that would elicit a more thorough provider explanation. Analyses of patients’ preferences resulted in elimination of 33 questions to yield a 34-item CKD-QPS.

### Refinement step 1: 6-person multidisciplinary team review

Next, the list was reviewed by the 6-person multidisciplinary research team to assess question wording, repetitiveness, and anticipated provider responses. This step served to optimize readability and ensure that each question would elicit a meaningful provider response. Two questions were eliminated by the team, yielding a 32-item QPS.

### Refinement step 2: readability assessment

The readability of the QPS overall and specific items was assessed using two online tools (Gunning Fog Index, and Flesch-Kincaid Grade Level Score) estimating the amount of formal education required to comprehend the printed material [32].

### Refinement step 3: feedback from the National Kidney Disease Education Program (NKDEP) Coordinating Panel

To further validate the instrument, the QPS was then reviewed by the NKDEP Coordinating Panel, which consists of individuals who are actively engaged in improving CKD detection and treatment. Based on their expert opinion, we further revised item wording and eliminated repetitive questions. One question that was previously removed from the list due to low rating (“How can I have kidney disease when I feel fine and make a lot of urine?”) was rephrased and re-inserted (“How can I have kidney disease when I feel fine?”). Two dialysis-related questions (“How long will it be before I need dialysis and transplant?” and “When do I have to start dialysis?”) were combined into one item (“Will I need dialysis and transplant? When?”). Another question that scored highly (“How can I get a kidney transplant outside of the VA?”) was deleted to improve generalizability of the QPS. The final QPS included 31-items.

## Results

### Participant characteristics

Four hundred forty-six patients were invited to participate and 213 participants were reached by phone. Of those, 18 were ineligible and among the 195 individuals, 68 were not interested, 62 did not schedule an interview, and 85 participated (Phase 1 = 32, Phase 2 = 53) (participation rate 44 %). Patients who declined participation reported that they were: not interested, did not have time, or did not want to do a telephone interview.

Participants were primarily male (97 %) and white (71 %), with a mean age of 67 years. CKD stages 3–5, and those on dialysis or with a transplant were represented as follows: CKD-3 (eGFR 30–59 ml/min/1.73 m^2^) (42 %), CKD-4 (eGFR 15–29 ml/min/1.73 m^2^) (25 %), CKD-5 (eGFR <15 ml/min/1.73 m^2^) (9 %), end-stage kidney disease (ESKD) on dialysis (8 %), and kidney transplant recipient (25 %). Demographic and clinical characteristics are represented in Table 1.

**Table 1:** Demographic and Clinical Characteristics of Study Participants

Characteristic	Phase 1 *N* (%)	Phase 2 *N* (%)	Total *N* (%)
Number Participants	32 (37.6)	53 (62.4)	85 (100)
Mean age in years	63 ± 9.55	69 ± 6.9	67 ± 8.8
Gender			
Male	30 (93.7)	52 (98.1)	82 (96.5)
Female	2 (6.3)	1 (1.9)	3 (3.5)
Race/Ethnicity			
Non-Hispanic White	16 (50.0)	44 (83.0)	60 (70.6)
African American	12 (37.5)	6 (11.3)	18 (21.2)
Hispanic-White	4 (12.5)	3 (5.7)	7 (8.2)
Marital Status			
Married	15 (46.9)	29 (54.7)	44 (51.8)
Not Married	17 (53.1)	24 (45.3)	41 (48.2)
Employment			
Employed	3 (9.4)	2 (3.8)	5 (5.9)
Unemployed	29 (90.6)	51 (96.2)	80 (94.1)
Annual Income^a^			
< 30,000	17 (53.1)	15 (28.3)	32 (37.6)
≥ 30,000	12 (37.5)	37 (69.8)	49 (57.6)
Highest Level of Education			
Less than High School	1 (3.1)	2 (3.8)	3 (3.5)
High School/GED	7 (21.9)	18 (34.0)	25 (29.4)
Some College	17 (53.1)	20 (37.7)	37 (43.5)
College Graduate	7 (21.9)	13 (24.5)	20 (23.5)
CKD Severity			
CKD-3	11 (34.4)	25 (47.2)	36 (42.4)
CKD-4	8 (25.0)	13 (24.5)	21 (24.7)
CKD-5	5 (15.6)	3 (5.7)	8 (9.4)
ESKD on dialysis	4 (12.5)	3 (5.7)	7 (8.2)
Kidney transplant recipient	4 (12.5)	9 (17.0)	13 (24.5)
Overall Health Status			
Excellent	0 (0)	4 (7.5)	4 (4.7)
Very Good	4 (12.5)	8 (15.1)	12 (14.1)
Good	15 (46.8)	26 (49.1)	41 (48.2)
Fair	6 (18.8)	11 (20.8)	17 (20.0)
Poor	7 (21.9)	4 (7.5)	11 (12.9)

^a^Percent does not add up to 100 because four participants (Phase 1 = 3; Phase 2 = 1) did not answer

### Patients’ unmet information needs

Patients reported having unmet information needs pertaining to all aspects their CKD. Two key CKD topics that patients desired the most information about included: treatment options for kidney failure (dialysis, kidney transplant), and the relationship between CKD and other chronic medical conditions. During Phase 1 interviews, regardless of patient’s CKD stage, most patients desired receiving additional information about dialysis and kidney transplantation (74 %) and reported that they wanted providers to disclose treatment options earlier in their course of care (72 %). The following quotations highlight patients’ desire for early education about ESKD treatment options:

“The moment they tell you what it [CKD] is! As soon as you see the kidney doctor! Ignorance is the worst. Tell me what can happen: ‘you have kidney disease. Down the road, you may need dialysis – there’s hemodialysis, peritoneal dialysis, or may not need it at all’”. (ID#6013, Kidney transplant recipient)

“This coming Monday! I have an appointment with my doctor. I’d want to know about this stuff now. I mean learning that I was a diabetic was ‘life changing’. I want to know about it early because it might change my life”. (ID#4014, CKD-4)

During Phase 1 interviews, patients commonly reported having co-existing medical conditions and desired information about how these conditions were related. Patients reported difficulty comprehending how their medical conditions related to one another and did not realize that CKD was associated with other chronic health problems (e.g., anemia, bone disease). The quotations below highlight patients’ desire to understand the relationship between their health problems:

“I did want to know how my diabetes affected my kidneys. I still don’t understand that. They just keep telling me that it was the diabetes but I don’t get how”. (ID#3007, CKD-3)

“I didn’t think the kidneys affected other parts of my body. I do want to know this”. (ID#4014, CKD-4)

“Why kidneys cause bone disease – makes no sense to the lay person”. (ID#5018, CKD-5)

After completing Phase 2 interviews, the two highest rated questions (that have since been minimally revised), referred to managing chronic conditions: “How do my CKD and other health problems (i.e., diabetes, hypertension, heart disease) affect each other?” and “What health problems can kidney disease cause?”).

### Patients desired a CKD-QPS

Most patients (88 %) reported that they were ‘very’ or ‘completely’ willing to use a CKD-QPS. Patients offered the following comments in support of a CKD-QPS:

“The mind is tricky. Sometimes you can’t think, especially when afraid. Having a list of questions will help remember what you wanted to ask”. (ID#5002, CKD-5)

“… I wish my primary doctor had given me a question list before my initial nephrology visit. Often patients are overwhelmed when they have to see the specialists, and I didn’t know what questions I should ask…” (ID#3042, CKD-3)

Only ten patients were not ‘very’ or ‘completely’ willing to use the QPS. Some of these patients offered a rationale for their reluctance, including their: desire to first evaluate the final QPS, ability to remember their own questions, or ability to “handle it all” without a QPS. Despite patients’ enthusiasm for using a QPS, only 35 % of patients reported making their own list of questions in preparation for healthcare visits.

### Desire for different CKD-QPSs

Given the broad range of CKD knowledge that patients are expected to gain throughout their disease course, Phase 2 participants were asked whether they preferred one comprehensive CKD-QPS or different lists tailored to CKD severity. Patients were slightly more in favor of having two separate lists (e.g., one for general CKD questions and one for dialysis/kidney transplant) (55 %) over one comprehensive list (40 %). However, participants who favored two lists reckoned that all patients still needed to be aware of renal replacement therapies, and stated for example:

“…but you do need to warn them about dialysis and transplant, so keep 1–2 questions about dialysis or transplant on the list”. (ID#3180, CKD-3)

Those who favored one list offered the following rationale:

“I like 1 big list; know what’s going on and what’s going to happen; what you are in for. Tell me so that it doesn’t surprise me”. (ID#4140, CKD-4)

### The final QPS

The final, 31-item QPS is presented in Table 2. The QPS was divided into 7 topics: 1) what is CKD; 2) impact of CKD on my life; 3) monitoring CKD; 4) self-care management; 5) treatment for kidney failure: general; 6) treatment: dialysis; and 7) treatment: kidney transplant.

**Table 2:** Final CKD-QPS

Many people with chronic kidney disease (CKD) have questions or concerns that they want to discuss with their healthcare team. During busy clinic visits people may forget to ask their questions. We created this question sheet to help you get the information that you want about your CKD.
The questions on this list are organized by topic. Some questions may matter more to you than others. You can use this list to help you remember what to ask your healthcare team. Circle the questions that you want answers to or write down your own questions before your clinic visit. Plan to ask your most important questions first. One visit may not be long enough to cover all of your questions.
Topics and Questions
What is CKD
What is chronic kidney disease (CKD)?
Is my CKD going to get worse?
What caused my CKD?
Impact of CKD on My Life
How does CKD affect my day-to-day life?
How do my CKD and other health problems (i.e., diabetes, high blood pressure, heart disease) affect each other?
Is my blood pressure where it should be?
What health problems can kidney disease cause?
What happens if my kidneys stop working?
Monitoring CKD
How can I have CKD when I feel fine?
What are the symptoms of CKD?
How do I know if my CKD is getting worse?
How much function is left in my kidneys now?
What is percent kidney function (GFR)? What is creatinine? What is urine protein?
Self-Care Management
What can I do to keep my kidney disease from getting worse?
What foods should I avoid?
What fluids should I avoid?
How much fluid should I drink each day?
What over the counter medicines should I avoid?
What medicines can I take to treat my kidney disease?
Treatment for Kidney Failure: General
What are all of the treatments for kidney failure?
Will I need dialysis or kidney transplant? When?
How long do patients live on dialysis versus with a transplant?
What will my life be like on dialysis versus with a transplant?
What happens if I do not do dialysis or get a transplant?
Treatment: Dialysis
What is dialysis? How does it work?
What is the difference between hemodialysis and peritoneal dialysis?
How will dialysis make me feel? Is dialysis painful?
Treatment: Kidney Transplant
How do I get a kidney transplant?
How long will a transplant last?
What kinds of medicine will I have to take after kidney transplant?
What is the surgery like for transplant?
Your own questions:

Based on the readability assessments, the final overall QPS scored at a 4^th^ to 5^th^ grade reading level. Few individual items scored higher because they included multisyllabic, albeit essential, CKD-related terminology (e.g., peritoneal dialysis, hemodialysis).

## Discussion

In this study, we developed a 31-item QPS targeted to patients with moderate CKD to facilitate their engagement in healthcare conversations. In the process of QPS development, CKD patients reported having unmet CKD information needs, corroborating findings from other studies [7, 21], and indicated that they wanted to use a CKD-QPS during their healthcare visits. Our CKD-QPS may facilitate patients’ involvement in healthcare discussions by teaching them to communicate their questions and concerns, thereby influencing providers to give patient-centered explanations [33, 34]. To our knowledge, this is the first QPS developed for the CKD population. We targeted the QPS to CKD patients with moderate disease in order to allow time to potentially attenuate CKD progression and improve intermediate and long-term health outcomes among a large patient population.

We envision that CKD patients will use this QPS with primary care providers prior to nephrology referral and during initial specialty care visits with their nephrologists. Use of the CKD-QPS in the primary care setting may facilitate timely referral to and prepare patients for nephrology care [24]. The QPS includes questions addressing general CKD information needs (e.g., cause of CKD, self-care management, impact on my life) with fewer questions pertaining to dialysis or transplantation. This inclusive approach was taken as patients learn about their CKD at different stages of disease severity, with varying levels of CKD knowledge and information needs. Further, this QPS accommodates patients’ reported desire to learn about renal replacement therapy early on, regardless of whether the treatment would be necessary. However, limiting the number of QPS questions is necessary because evidence suggests that longer QPSs may increase length of clinical encounter time [13]. Therefore, our CKD-QPS does not include an exhaustive list of CKD-related questions, but encourages patients to develop their own additional questions.

Patients who are more involved in their healthcare have better outcomes including more preventative care, decreased hospitalizations, improvement in disease-specific outcomes, and greater patient satisfaction [35, 36]. QPS interventions enable patients to embrace a more active role when communicating with providers [37–39], and may improve their disease knowledge, and better equip them with the skills to confidently participate in their healthcare. Investigators recognize the need for active patient communication and have thus developed QPSs in other chronically ill groups (e.g., cancer, primary care, pediatric attention deficit-hyperactivity disorder, and pre-surgery). The effect of QPS interventions on long-term health outcomes has not been studied. Some studies have shown that question-asking interventions improved patients’ satisfaction [16], increased the number of questions-asked [37, 38, 40], enhanced post-visit recall [37, 39], and did not increase in length of clinical encounter [39, 41]. However, meta-analyses have reported inconclusive results regarding the association between QPS usage and communication outcomes [13, 15, 42, 43]. Because the field is in its nascence and studies vary greatly in regards to QPS characteristics and measured outcomes, caution should be exercised in the interpretation of the existing literature [13, 15]. Future research is needed to identify the optimal mode of distributing the CKD-QPS and to assess the effects of the QPS on short-term (e.g., patient-provider communication, question-asking, patient satisfaction, length of clinic visit), intermediate (e.g., patients’ CKD knowledge and recall, adherence to self-care practices and medications), and long-term outcomes (e.g., ESKD treatment planning, comorbidity control, hospitalizations, and CKD progression). Future studies with larger sample sizes in both VA and non-VA populations are needed to characterize patients’ question-asking based on CKD severity and other socio-demographic characteristics, improve generalizability, and further refine the CKD-QPS.

Strengths of this study include use of a patient-centered approach that prioritized patients’ preferences above other stakeholders’ input. Similar to other QPS studies, we adopted a rigorous mixed methods approach to data collection and analysis, and a multi-stage process with multidisciplinary team input. Further, input from the NKDEP Coordinating Panel supported the face validity of QPS items. Moreover, the CKD-QPS was developed at a <5^th^ grade reading level, which corresponds with the Institute of Medicine recommendation that printed health-related information not exceed a 6^th^ grade reading level [44]. Given that universal health literacy precautions were used, patients at all health literacy levels can use this QPS. This reading level is important given the high prevalence of inadequate health literacy in the general public [44], and especially in the CKD patient population [45].

This study has limitations. First, the patient population was derived from one VA nephrology center, which may limit generalizability of findings. The Veteran population is characteristically elderly, male, and white. While these demographic characteristics are representative of the majority of ESKD patients in the USA [1], we attempted to oversample for minority groups and female patients to ensure representation of all patients’ CKD information needs. Fifty percent of phase 1 participants were either African American or Hispanic-White, reflecting representation of a diverse minority population; however, the heavily male VA population restricted our ability to recruit women. Additionally, approximately a quarter of our study participants were kidney transplant recipients. As kidney transplant recipients are generally more activated than the general CKD population, their perspectives may have overly influenced the questions included in the QPS. Conversely, patients who were unaware of their kidney disease were excluded from participation, and therefore their information needs were not assessed or reflected in the CKD-QPS. We obtained feedback from the NKDEP Coordinating Panel to help overcome these limitations and improve the generalizability of the QPS in non-VA settings. Second, there were some discrepancies between the quantitative ratings of QPS items and the qualitative feedback. Whereas most QPS items were rated highly, resulting in a ceiling effect, the qualitative feedback provided valuable context for interpreting those ratings. Lastly, our findings are subject to recall bias, as with any cross-sectional study, and to interviewers’ bias as interviews were not audio-recorded.

## Conclusions

We developed a 31-item QPS targeted to patients with moderate CKD to address their unmet information needs, engage patients in healthcare discussions, and facilitate patient-centered care. The CKD-QPS has tremendous potential to improve patients’ CKD knowledge, patient-provider communication, and health outcomes. Future research should assess the impact of the CKD-QPS on short-term, intermediate, and long-term health outcomes.

## Additional file

Additional file 1: CKD-QPS Development: Phase 1 Interview Guide. (DOCX 24 kb)

## Abbreviations

CKD: Chronic kidney disease
eGFR: Estimated glomerular filtration rate
ESKD: End-stage kidney disease
IRB: Institutional review board
NKDEP: National Kidney Disease Education Program
QPS: Question prompt sheet
VA: United States Department of Veteran Affairs
