# Supplementary material for: A question prompt sheet for adult patients with chronic kidney disease
Source: BMC Nephrol. 2016 Oct 19;17:155. doi: 10.1186/s12882-016-0362-z (PMC5070305; doi:10.1186/s12882-016-0362-z)
Supplement: Additional file 1: — CKD-QPS Development: Phase 1 Interview Guide. (DOCX 24 kb) [file 12882_2016_362_MOESM1_ESM.docx]

**CKD-QPS Development: Phase 1 Interview Guide**

**Six Item Screener (SIS)**

*I would like to ask you some questions that ask you to use your memory. I am going to name three objects. Please wait until I say all three words, then repeat them. Remember what they are because I am going to ask you to name them again in a few minutes. Please repeat these words for me: APPLE-TABLE-PENNY.* (Interviewer may repeat names 3 times if necessary but repetition not scored.)

**Did patient correctly repeat all three words?** *Yes No*

Incorrect Correct

1. What year is this? 0 1
2. What month is this? 0 1
3. What is the day of the week? 0 1

**What were the three objects I asked you to remember?**

1. Apple = 0 1
2. Table = 0 1
3. Penny = 0 1

**Patient’s CKD Awareness**

*Now, I’d like you to think back to the last time you saw a kidney doctor.*

**1. Do you remember going to a kidney doctor (or your renal doctor, or nephrologist)? [Intention: Find out if patients are aware that they have CKD]**

___ Yes

___ No

**2. What has your kidney/renal doctor (nephrologist) told you about your kidney disease/function? How well are your kidneys are doing? [Intention: to produce more patient-centered language to use for QPS questions]**

**3. Is patient aware of having CKD?**

___ Yes: Q1 = Yes or No + Q2 = Awareness 🡪 Proceed with interview

___ No: Q1 = No + Q2 = Unaware 🡪 Stop interview

1. ***Now let’s talk about the information you’d like to find out about chronic kidney disease or “CKD”. I’m going to give you a series of prompts. For each prompt, I want you to answer 2 questions, 1) what do (did) you want to know about it and 2) What questions you have about it.***

**Prompt A:** “your chronic kidney disease.” Tell me what kinds of things you want to know about this and what kinds of questions you have…

**Prompt B:** “How can you tell that you have CKD.” Tell me what you want to know about this and what kinds of questions you have…

**Prompt C:** “What caused your CKD.” What do you want to know about that, what kinds of questions you have about what caused your kidney disease…

**Prompt D:** “How do you know if your CKD is getting worse.” (PROBE: how your CKD is being tracked/followed.) What do you want to know about this? What kinds of questions have you had about how your CKD is being tracked/followed?…

**Prompt E:** “What treatments or therapies can your doctor give you to make CKD get better.” What kinds of things do you want to know about this and what kinds of questions do you have…

**Prompt F:** “What treatments or therapies can your doctor give you to prevent CKD from getting worse.” What do you want to know about this and what kinds of questions do you have…

**Prompt G:** “Other things that you can do to manage your CKD.” What do you want to know about that?

**PROBE:** What kinds of questions do you have about …

1. What non-prescription meds can help or harm kidney
2. What vitamins/herbals can help or harm kidney
3. Other lifestyle choices (exercise, water) can help your kidneys

**Prompt H:** “What other health conditions or problems (ie DM/HTN) can affect your CKD.” What kinds of things did you want to know about this and what kinds of questions do you have…

**Prompt I**: “How other health conditions can affect your CKD.” What do you want know about this and what kinds of questions do you have…

**Prompt J:** “What the complications of CKD are (ie. Anemia, bone dz 🡪prognosis)”. What do you want to know about that, and what kinds of questions do you have…

**Prompt K:** “What do you want to know about dialysis or kidney transplant.” What kinds of questions do you have…

**Prompt L:** “When do you think is the right time to learn about dialysis or kidney transplant.” What kinds of questions do you have…

1. ***OK great. Thank you for this information. I have a few more questions related to your information needs and then I’ll ask you a few more questions about yourself.***

What do you wish you had asked before you got CKD? Or, that you has asked before your CKD got worse?

What information is most important to know about chronic kidney disease?

Assuming that you trust your doctor and have a great relationship w/ him or her, what questions would

you ask about CKD (in order to get the information you need) (PROBE – was there information that you

wanted from your doctor a/b CKD)

1. **Making lists is common. Some people make grocery lists or to-do lists. Have you ever made a list to use in a doctor visit? For example, some people make a medication list.**

___Yes 🡪 IF YES, what kind of list have you made?

___ No

**4. Have you ever made a list of questions?**

___Yes 🡪 IF YES, would you be willing to share your list?

___ No

**5. How willing would you be to use a question list about CKD in your doctor visits?**

___ Not at all willing

___ A little willing

___ Somewhat willing

___ Very willing

___ Completely willing

**Demographics**

*We have a few questions to ask you about your background.*

**1. Gender:**

___ Male

___ Female

**2. What is your age?**

___ Code age in years

**3. Are you Hispanic or Latino?**

___ Yes

___ No

**4. Which one or more of the following would you say is your race? (Check all that apply) Please read:**

___ White

___ Black or African American

___ Asian

___ Native Hawaiian or Other Pacific Islander

___ American Indian or Alaska Native

___ Other [specify]______________

**5. What is your marital status?**

___ Married

___ Separated

___ Divorced

___ Widowed

___ Never married

___ A member of an unmarried couple

**6. What is the highest grade or year of school you completed?**

___ Never attended school or only attended kindergarten

___ Grades 1 through 8 (Elementary)

___ Grades 9 through 11 (Some high school)

___ Grade 12 or GED (High school graduate)

___ College 1 year to 3 years (Some college or technical school)

___ College 4 years or more (College graduate)

**7. Are you currently…?**

___ Employed for wages (full or part time)

___ Unemployed

___ A Homemaker

___ A Student

___ Retired

___ Unable to work

**8. The next question asks about your income. This information is important for understanding how finances can affect how patients manage their health. So, what was your total annual household income from all sources in the past 12 months:**

___ Less than $15,000

___ Between $15,001 and $30,000

___ Between $30,001 and $45,000

___ Between $45,001 and $60,000

___ Between $60,001 and $75,000

___ Between $75,001 and $90,000

___ More than $90,001

**9a. In general, in what language do you prefer to talk to health providers about your medical care?**

___ English

___ Spanish

___ English and Spanish equally

**9b. How well do you speak English?**

___ Not at all

___ Not well

___ Well

___ Very well

**10. In general, would you say your health is:**

___ Excellent

___ Very good

___ Good

___ Fair

___ Poor

**11. If you are on hemodialysis, how long have you been on it?**

**12. If you have had a kidney transplant, how long have you had it?**

**13. Do you have any questions or comments?**

**Thank you for your time and your help today!**
